# Shoutai pills for threatened abortion: A protocol for systematic review and meta-analysis

**DOI:** 10.1097/MD.0000000000033173

**Published:** 2023-03-17

**Authors:** Chuangxiu Song, Shan Zhang, Xiaojing Gao, Haidi Zhang, Songbo Zuo, Yuxuan Qin, Xiaotao Bi, Huijuan Chen

**Affiliations:** a Hebei University of Traditional Chinese Medicine, Hebei Province, China; b Kailuan General Hospital of Tangshan City, Hebei Province, China; c Beijing General Hospital of Coal Industry Group, Beijing, China; d The Second Outpatient Department of Hebei Province, Hebei Province, China; e The First Affiliated Hospital of Hebei College of Traditional Chinese Medicine, Shijiazhuang, China.

**Keywords:** dydrogesterone tablets, meta-analysis, shoutai pills, systematic review, threatened abortion

## Abstract

Threatened abortions are a serious health risk for women. Deferiprone tablets are commonly used in the treatment of clinical delivery. Traditional Chinese medicine, a characteristic medical system inherited for thousands of years, often applies Shoutai pills in the treatment of Threatened abortion and has achieved good results. This systematic review and meta-analysis aimed to evaluate the efficacy and safety of Shoutai pills combined with dedrogesterone tablets for the treatment of early preterm abortion.

**Methods:**

Electronic searches of clinical randomized controlled trials in PubMed, Web of Science, MEDLINE, EMBASE, China National Knowledge Infrastructure, Wanfang database, and China Scientific Journal Database (VIP) were conducted. References to the included literature, gray literature in Open Grey, and other relevant literature such as clinical studies registered in ClinicalTrials.gov, were also manually searched. Relevant data were extracted, and a meta-analysis was performed using Reviewer Manager 5.4.

**Results:**

The results of this study will be submitted to peer-reviewed journals.

**Conclusion:**

This study provides high-quality evidence on the efficacy and safety of Shoutai pills in combination with dedrogesterone tablets for the treatment of preterm abortion.

## 1. Introduction

Threatened abortion (TA) is a condition that occurs before 28 weeks of gestation with vaginal bleeding and lower abdominal pain and can cause anxiety and depression in women and even miscarriage,^[1]^ which is a common complication of pregnancy, with a prevalence of 10%. TA is associated with fetal chromosomal abnormalities, age, and endocrine status. Living conditions and men’s health are among the factors contributing to this condition.^[2]^

Currently, there are no clear clinical options for the treatment. Expectant therapy, pharmacotherapy, or surgical therapy are commonly used treatments.^[3]^ Among these, progesterone support therapy is considered one of the most effective and necessary therapies to establish an adequate immune response to prevent miscarriage.^[4]^ Didrogestrel tablets (Dt) are a relatively new type of progesterone that specifically binds to the progesterone receptor and has no significant teratogenic effects. In addition, it is characterized by rapid absorption and few adverse gastrointestinal effects. However, the clinical efficacy of Dt for fetal protection needs to be improved owing to large individual differences. Therefore, there is a need to develop alternative therapies and management strategies for TA.^[5–8]^

In recent years, Chinese medicine has gained considerable attention in the treatment of TA. Shoutai pills (St) are a typical Chinese herbal formula invented by Zhang Xichun and consist of 4 natural herbs: Cuscuta sinensis, Sambucus nigra, Chuan Xue Guan, and Agaricus blazei.^[9]^ Previous studies have shown that St can effectively promote kidney notification and the maintenance of pregnancy, with excellent results in the treatment of TA.^[10–13]^ However, the small sample sizes of these clinical trials did not allow for a systematic evaluation of the specifics of St in the treatment of preterm abortion.

In this study, we analyzed the efficacy and safety of St combined with Dt in the treatment of preterm abortion.^[14,15]^ By comparing the clinical data of St combined with Dt and Dt alone, we will explore the specifics of this regimen in a clinical setting and provide a reference for clinical treatment.

## 2. Materials and methods

This study was analyzed according to the Preferred Reporting Items for Systematic Evaluation and Meta-Analysis (PRISMA) manual, and the review will be conducted according to Preferred Reporting Items for Systematic Evaluation and Meta-Analysis filing guidelines. The study was registered with PROSPERO (registration number: CRD42022382559). This study was based on the published literature and did not require ethical approval.

### 2.1. Types of studies

The randomized controlled clinical trials included were not restricted by region or language. Reviews, non-randomized clinical trials, animal studies, and studies lacking experimental data were excluded.

### 2.2. Types of participants

All patients had a clear diagnosis of preterm abortion, with no restrictions on nationality, age, race, or etiology.

### 2.3. Types of intervention

The test group was treated with oral Dt in combination with Shou Fei Pills. The dose and frequency of Dt, and the dosage form and dose of St were not restricted. The control group received the Dt intervention only, and the dose and frequency of Dt were not restricted. Meanwhile, patients in both groups did not receive any other treatment.

### 2.4. Types of outcome measures

#### 2.4.1. Main outcome.

The purpose of this study was to investigate the effectiveness and safety of St in TA. Therefore, we chose the total effective rate as the main outcome of this study. In addition, some relevant indicators can be included in the study outcome, including gynecological ultrasound, progesterone levels, and serum human chorionic gonadotropin levels.

#### 2.4.2. Secondary outcomes.

The secondary outcomes include

1. Traditional Chinese medicine symptom scores.
2. Pregnancy outcome (clinical pregnancy, persistent pregnancy, and early miscarriage).
3. Symptom scores, and.
4. anxiety and depression scores.

### 2.5. Literatures search

#### 2.5.1. Literatures search sources.

We searched the following databases: PubMed, Cochrane Library, Web of Science, Embase, MEDLINE, China Biology Medicine, Scopus, China National Knowledge Infrastructure, VIP, and Wanfang. We also searched the references of included articles, gray literature in OpenGrey, and other relevant literature, such as clinical studies registered in ClinicalTrials.gov. For those clinical randomized controlled trials that are ongoing or unpublished, the authors will be contacted for the most recent experimental data. All clinical databases are currently available on December 8 2022.

#### 2.5.2. Literature search strategy.

We used “Threatened Abortion” “Threatened Abortions” “Threatened Miscarriage” “Miscarriage, Threatened” “Threatened Miscarriages” “Dydrogesterone Tablets” “Isopregnenone” “6-Dehydro-9 beta-10 alpha-progesterone” “6 Dehydro 9 beta 10 alpha progesterone” “Dehydrogesterone” “Duphaston” “Shoutai Pills” “randomized controlled trial” “randomized” “placebo” and the like were used as keywords for the search. the specific search strategy is shown The specific search strategy is shown in Table 1 (PubMed was used as an example).

**Table 1:** Search strategy used in PubMed database.

Number	Search terms
#1	“Threatened Abortion” [Mesh]
#2	Threatened Abortions [Title/Abstract]
#3	Threatened Miscarriage [Title/Abstract]
#4	Miscarriage, Threatened [Title/Abstract]
#5	Threatened Miscarriages [Title/Abstract]
#6	#1 or #2 or #3 or #4 or #5
#7	“Dydrogesterone” [Mesh]
#8	Dydrogesterone Tablets [Title/Abstract]
#9	Isopregnenone [Title/Abstract]
#10	6-Dehydro-9 beta-10 alpha-progesterone [Title/Abstract]
#11	6 Dehydro 9 beta 10 alpha progesterone [Title/Abstract]
#12	Duphaston [Title/Abstract]
#13	#7 or #8 or #9 or #10 or #11 or #12
#14	“Shou Tai pills” [Mesh]
#15	STP [Title/Abstract]
#16	Shou Tai [Title/Abstract]
#17	#14 or #15 or #16
#18	randomized controlled trial [Publication Type]
#19	Randomized [Title/Abstract]
#20	Placebo [Title/Abstract]
#21	#17 or #18 or #19
#22	#6 and #13 and #17 and #20

### 2.6. Literature collection and organization

**Figure 1.**
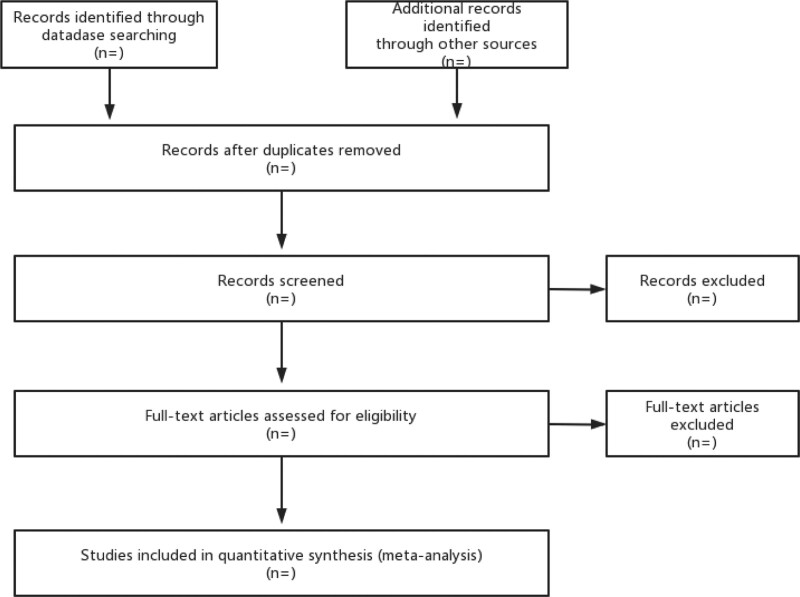
Flow chart of literatures screening.

Data collection and organization were performed separately by 2 researchers. EndNote was used to manage relevant literature and remove duplicates. Two researchers screened the literature by reading the titles, abstracts, and keywords of the articles and recorded the reasons for exclusion. When 2 researchers disagree, a third researcher will make a decision. The process is illustrated in Figure 1.

### 2.7. Data extraction and export

Two researchers extracted data from the literature and created an Excel spreadsheet. Valid information extracted included the title, author, year, disease diagnosis, sample size, age, intervention method, treatment duration, and outcome indicators. Disagreement were resolved by 2 investigators, and if necessary, a third investigator was invited to participate in the decision-making process.

### 2.8. Assessment of risk of bias

To assess the methodological quality of the included studies, 2 investigators independently assessed the risk of bias using the Cochrane tool for the included literature. Each study was categorized as having high, low, or unclear risk based on the following items: random sequence generation, allocation concealment, subject and personnel blinding, blinding for outcome assessment, incomplete outcome data, selective reporting, and other biases. scores 1 to 3 were categorized as high risk and scores 4 to 7 were categorized as low risk. In cases of disagreement, a third investigator determined the process.

### 2.9. Assessment of heterogeneity

The heterogeneity of information was assessed using the chi-square test and *I^2^* tests. When heterogeneity was not significant (*P* ≥ .10, *I^2^* ≤ 50%), a fixed-effects model was used for the analysis; when heterogeneity was significant (*I^2^* > 50% or *P* < .10), a random effects model was used.

### 2.10. Assessment of reporting biases

Reporting bias will be assessed when necessary to ensure the accuracy of the study results. If more than ten papers were included in this study, the symmetry of the funnel plot was assessed using Stata 14.0.

### 2.11. Data synthesis

Two separate meta-analyses were performed using Reviewer Manager version 5.4. Using 95% confidence intervals, defined differences were calculated for continuous variables, and risk ratios were calculated for dichotomous variables. The heterogeneity of information was assessed using the chi-square and *I^2^* tests. When heterogeneity was not significant (*P* ≥ .10, *I^2^* ≤ 50%), a fixed-effects model was used for analysis. When heterogeneity was significant (*I^2^* > 50% or *P* < .10), a random effects model was used.

### 2.12. Subgroups analysis

Subgroup analysis was performed if there was significant heterogeneity in the data analysis results. This will be performed based on age, race, treatment period, sample size, or other factors that may affect the results.

### 2.13. Sensitivity analysis

To ensure the reliability of the findings, a sensitivity analysis was performed based on the method quality, sample size, and missing data. Data analysis and comparison were performed again to assess the reliability of the results.

### 2.14. Grading the quality of evidence

The data in this study were obtained from published literatur; therefore, ethical approval was not required. The results of this study will be submitted to peer-reviewed journals.

## 3. Discussion

Clinical evidence suggests that St combined with Dt has better efficacy in preterm abortion. However, there is a lack of evidence-based medical support for this therapy in the treatment of preterm abortions. Therefore, this study investigated the efficacy and safety of St combined with Dt for TA by analyzing data from clinical randomized controlled trials of ST combined with Dt for TA. The results of this study will provide a scientific basis for the clinical application of ST combined with Dt in the treatment of TA, and optimize the treatment plan for this disease. However, bias and significant heterogeneity may have occurred during the course of this study, which may have affected the reliability of the results.

## Acknowledgments

This work was supported by the Scientific Research Program of the Hebei Provincial Administration of Traditional Chinese Medicine (No:2021060).

## Author contributions

**Conceptualization:** Huijuan Chen, Chuangxiu Song.

**Data curation:** Shan Zhang, Haidi Zhang.

**Formal analysis:** Xiaojing Gao.

**Methodology:** Yuxuan Qin, Xiaotao Bi.

**Software:** Chuangxiu Song, Shan Zhang.

**Supervision:** Huijuan Chen.

**Writing – original draft:** Huijuan Chen, Xiaojing Gao, Haidi Zhang.

**Writing – review & editing:** Chuangxiu Song, Songbo Zuo.
